# *catena*-Poly[[bis­(2-methyl­pyrazine-κ*N*^4^)copper(II)]-di-μ-bromido]

**DOI:** 10.1107/S2414314626010758

**Published:** 2026-10-23

**Authors:** Robert T. Butcher, Brendan Twamley, Mark M. Turnbull

**Affiliations:** a Carlson School of Chemistry and Biochemistry, Clark University, 950 Main St., Worcester, MA 01610, USA; b Dept. of Chemistry, Trinity College Dublin, The University of Dublin, College Green, Dublin 2, Ireland; Benemérita Universidad Autónoma de Puebla, México

**Keywords:** crystal structure, copper(II), bihalide bridged chain, methyl­pyrazine

## Abstract

The reaction of 2-methyl­pyrazine with copper(II) bromide *via* gel diffusion yielded crystals of the title compound, [CuBr_2_(C_5_H_6_N_2_)_2_]_*n*_. The compound crystallizes in the monoclinic *P*2_1_/*n* space group and the metal atom (site symmetry 1) adopts a square-planar CuBr_2_N_2_ coordination geometry. Short Cu⋯Br contacts, nearly perpendicular to the Cu coordination plane, link the mol­ecules into chains propagating parallel to the *a*-axis direction, which are well separated by the bulk of the methyl­pyrazine ligands.

## Abstract 2

In the title polymer, mononuclear complexes are linked through Cu⋯Br contacts, nearly perpendicular to the Cu coordination plane.

## Graphical abstract

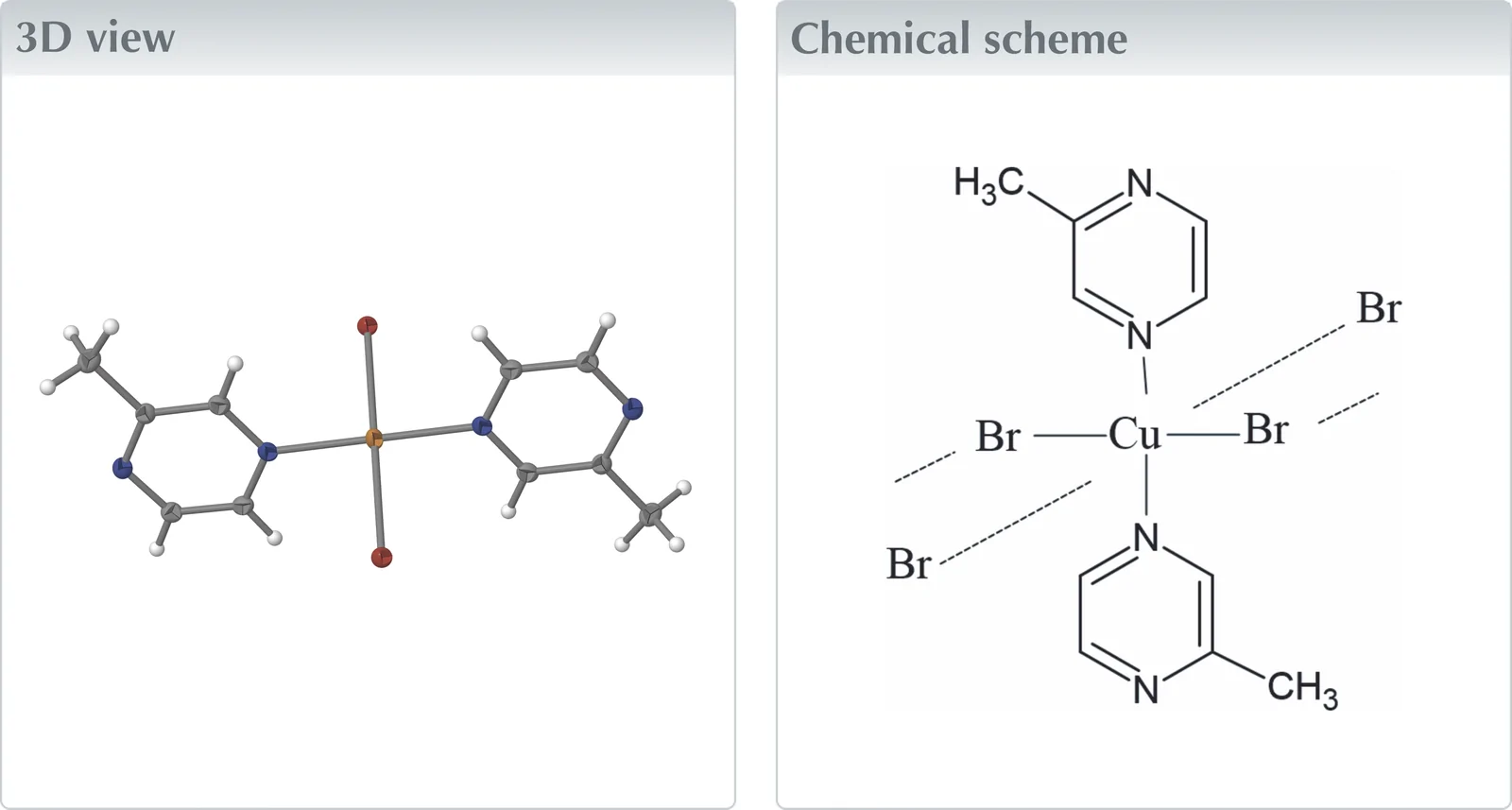

## Structure description

Pyrazine has been used ubiquitously in coordination chemistry, in particular with Cu^II^ (Landee & Turnbull, 2013 ▸). The two nitro­gen atoms provide a linear coordination bridge for good control of geometry in coordination polymers. Substituted pyrazine ligands have also been used frequently, but may introduce a change in local geometry depending upon the number and position of the substituents. In cases where the substituted pyrazine is monodentate, coordination occurs uniformly to the distal nitro­gen atom, except in cases where the substituent may coordinate in which case chelation is observed to the proximal nitro­gen atom, as in the case of pyrazine-2-carb­oxy­late (Klein *et al.*, 1981 ▸, 1982 ▸; Konar *et al.*, 2014 ▸).

2-Methyl­pyrazine is one of the most commonly employed monosubstituted pyrazine ligands with more than eighty complexes of first row transition metals alone; fifty of those involve copper (Groom *et al.*, 2016 ▸). Cu^II^ coordinates to methyl­pyrazine in both bridging (Otieno *et al.*, 1993 ▸; Abu-Youssef *et al.*, 2008 ▸) and terminal (Maekawa *et al.*, 1992 ▸; Navas *et al.*, 1993 ▸; Amaral *et al.*, 2001 ▸; Otieno *et al.*, 2002 ▸; Manson *et al.*, 2008 ▸; Barquín *et al.*, 2010 ▸; Araujo-Martinez *et al.*, 2023 ▸) modes, again with the terminal methyl­pyrazine mol­ecules coordinated through the distal nitro­gen atom. We find only three examples in the literature of terminal methyl­pyrazine mol­ecules coordinated through the proximal nitro­gen atom, namely bis­(μ-iodo)­bis­(2-methyl­pyrazine)­bis­(tri­phenyl­phosphine)dicopper(I) and its tri­phenyl­arsine equivalent (Kikuchi *et al.*, 2026 ▸), plus the silver(I) complex *catena*-[(μ2-methyl­pyrazine)­bis­(μ_2_-tri­cyano­methanide)bis­(methyl­pyrazine)­disilver(I)] (Abrahams *et al.*, 2003 ▸).

In the course of our investigations of complexes of substituted pyrazines with Cu^II^, we prepared the title compound, which crystallizes in the monoclinic space group *P*2_1_/*n* with the Cu^II^ ion located on an inversion centre. The mol­ecular unit is shown in Fig. 1 ▸. The Cu1—N14 bond length is 2.021 (2) Å (Table 1 ▸) and comparable to known compounds with methyl­pyrazine coordinated to the distal nitro­gen atom (Otieno *et al.*, 2002 ▸; Araujo-Martinez *et al.*, 2023 ▸) but somewhat shorter (∼0.05 Å) than the equivalent bond in compounds where the methyl­pyrazine is bridging (Otieno *et al.*, 1993 ▸). When bridging, it is common for the position of the methyl group to be disordered (Amaral *et al.*, 2001 ▸; Liu *et al.*, 2017 ▸; Kikuchi *et al.*, 2023 ▸), rendering the distal and proximal nitro­gen atoms equivalent and the bond length moot. The Br1—Cu1—N14 bond angle is 90° within error and all *trans* bond angles are 180° as required by symmetry. The pyrazine ring is virtually planar, as expected (mean deviation of constituent atoms = 0.006 Å) and the ring is canted 60.6 (2)° relative to the Cu coordination plane.

In the extended structure, the mol­ecular units are linked into chains parallel to the *a*-axis direction *via* short Cu⋯Br contacts forming bihalide bridges (see Fig. 2 ▸). The Cu1⋯Br1^i^ distance is 3.1912 (3) Å [symmetry code: (i) 2 − *x*, −*y*, 1 − *z*) and the Br1—Cu1—Br1^i^ angle is 92.141 (10)°. The Cu1—Br1⋯Cu1^ii^ angle supplementary [87.859 (10)°; symmetry code: (ii) 1 + *x*, *y*, *z*] as required by symmetry. The long Cu⋯Br contact lies only 2.2 (1)° from the normal to the Cu^II^ coordination plane. The chains are well separated by the bulk of the methyl­pyrazine ligands (see Fig. 3 ▸), suggesting that the chains would be well isolated magnetically. The structure of the bihalide bridges within the chains suggests weak anti­ferromagnetic inter­actions. Sadly, insufficient material was isolated to measure the magnetic properties.

## Synthesis and crystallization

Single crystals suitable for single-crystal X-ray diffraction were made by a gel diffusion technique. The bottom gel layer was prepared by dissolving copper(II) bromide (1.001 g, 4.49 mmol) and 1 ml of tetra­methyl orthosilicate (TMOS) in 9 ml of H_2_O. The mixture was stirred for 5 min. and allowed to harden overnight. The second layer was made by adjusting the pH of 18 ml H_2_O and 2 ml TMOS with 20 drops of 2% ammonium hydroxide. This mixture was carefully poured over the top of the first layer, and also allowed to harden overnight. The third layer was prepared by dissolving 2-methyl­pyrazine (375.8 mg, 3.99 mmol) and 1 ml of TMOS in 10 ml of H_2_O, stirring for 5 minutes, and pouring the solution over the top of the previous two layers. The layered gels were allowed to sit for 8 months yielding green needles approximately 1.10 × 0.06 × 0.04 mm in size on the surface of the gel. The crystals were harvested by filtration and washed with 30 ml of diethyl ether. IR (KBr) ν: 3083*w*, 1595*w*, 1482*w*, 1443*m*, 1400*m*, 1302*m*, 1259*w*, 1164*m*, 1081s, 1026*m*, 981*w*, 909*w*, 830*s*, 737*m*, 668*s*, 567*w*, 503*w* cm^−1^.

## Refinement

Crystal data, data collection and structure refinement details are summarized in Table 2 ▸. A very large crystal was used (1 mm long) due to the braided nature of the sample. Once cut, the de-braided end was kept out of the beam path. No decomposition was observed during data collection.

## Supplementary Material

Crystal structure: contains datablock(s) I. DOI: 10.1107/S2414314626010758/bh4105sup1.cif

Structure factors: contains datablock(s) I. DOI: 10.1107/S2414314626010758/bh4105Isup2.hkl

CCDC reference: 2595817

Additional supporting information:  crystallographic information; 3D view; checkCIF report

## Figures and Tables

**Figure 1:**
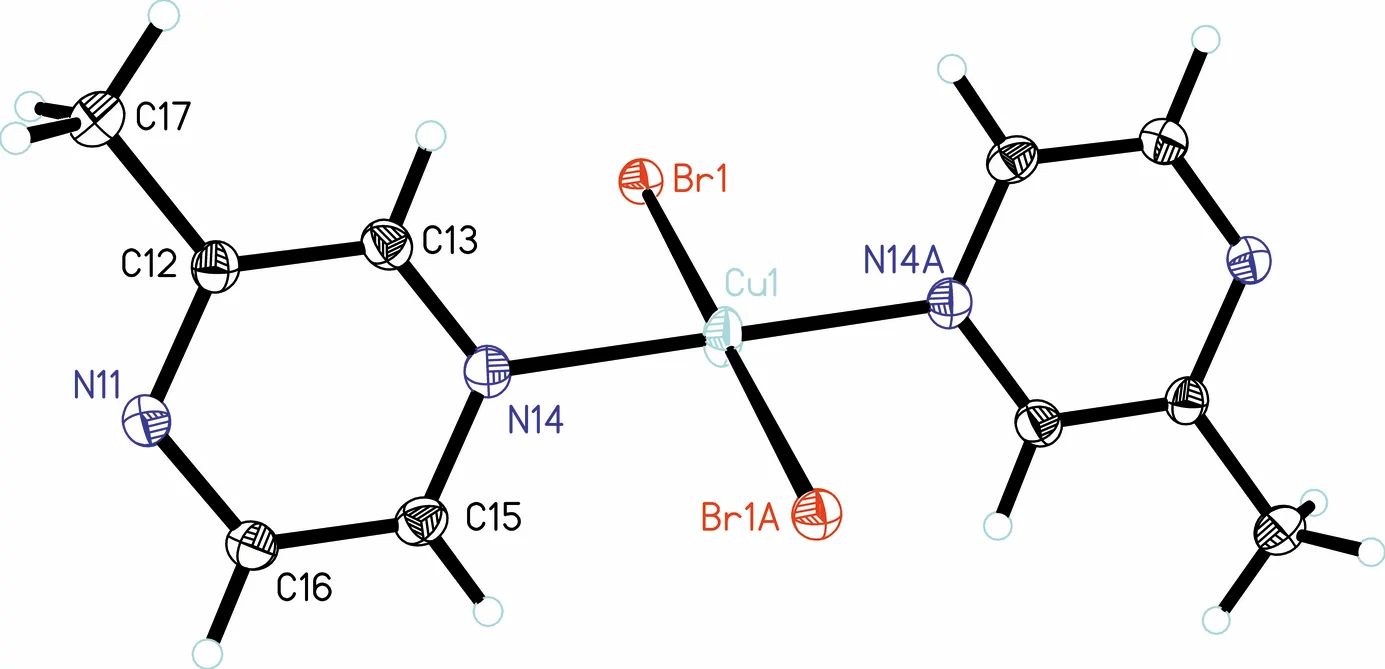
The mol­ecular unit drawn as 50% probability ellipsoids. Hydrogen atoms are shown as spheres of arbitrary size. Only the asymmetric unit and copper coordination sphere are labelled. Atoms with suffix A are generated by the symmetry operation −x + 1, −y, −z + 1.

**Figure 2:**
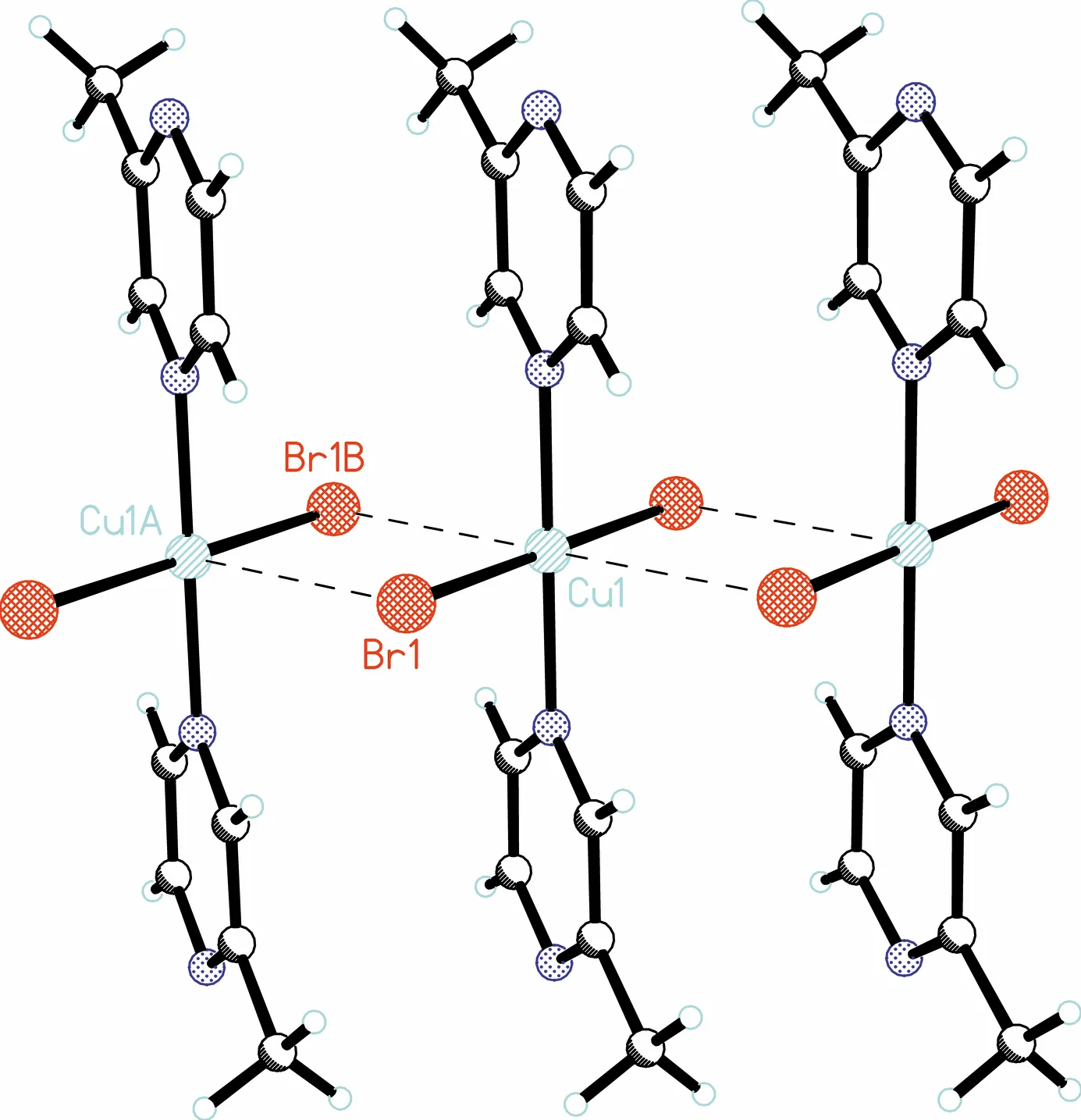
The chain structure of the compound shown as arbitrary spheres (*a* axis horizontal).

**Figure 3:**
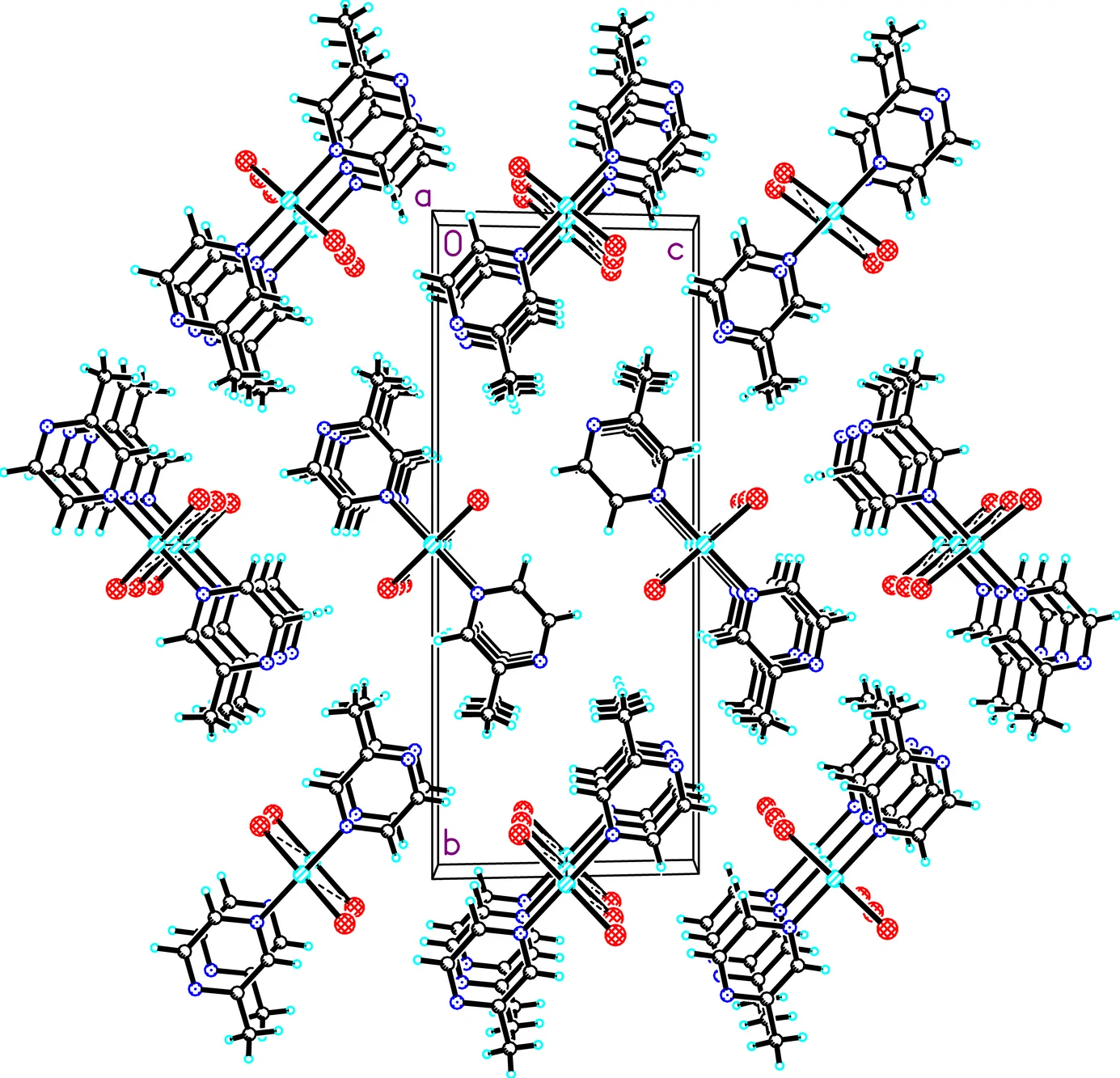
Packing of the chains viewed down the *a* axis. Atoms are shown as arbitrary spheres.

**Table 1:** Selected geometric parameters (Å, °)

Cu1—N14	2.021 (2)	Cu1—Br1	2.4196 (3)
			
N14—Cu1—Br1	89.84 (6)		

**Table 2:** Experimental details

Crystal data	
Chemical formula	[CuBr_2_(C_5_H_6_N_2_)_2_]
*M*_r_	411.60
Crystal system, space group	Monoclinic, *P*2_1_/*n*
Temperature (K)	90
*a*, *b*, *c* (Å)	3.9321 (4), 20.274 (2), 8.2685 (9)
β (°)	99.602 (2)
*V* (Å^3^)	649.93 (12)
*Z*	2
Radiation type	Mo *K*α
μ (mm^−1^)	7.81
Crystal size (mm)	1.10 × 0.06 × 0.04

Data collection	
Diffractometer	Bruker SMART APEX
Absorption correction	Multi-scan (*SADABS*; Krause *et al.*, 2015 ▸)
*T*_min_, *T*_max_	0.042, 0.730
No. of measured, independent and observed [*I* > 2σ(*I*)] reflections	8934, 1837, 1674
*R*_int_	0.042
(sin θ/λ)_max_ (Å^−1^)	0.703

Refinement	
*R*[*F*^2^ > 2σ(*F*^2^)], *wR*(*F*^2^), *S*	0.029, 0.074, 1.08
No. of reflections	1837
No. of parameters	80
H-atom treatment	H-atom parameters constrained
Δρ_max_, Δρ_min_ (e Å^−3^)	1.68, −0.62

Computer programs: *SMART* and *SAINT-Plus* (Bruker, 2006 ▸), *SHELXS2014* (Sheldrick, 2008 ▸), *SHELXL2019/2* (Sheldrick, 2015 ▸), *XP* (Bruker, 2004 ▸) and *publCIF* (Westrip, 2010 ▸).

## full crystallographic data

### catena-Poly[[bis(2-methylpyrazine-κN4)copper(II)]-di-µ-bromido] Crystal data

**Table:**

[CuBr_2_(C_5_H_6_N_2_)_2_]	*F*(000) = 398
*M**_r_* = 411.60	*D*_x_ = 2.103 Mg m^−^^3^
Monoclinic, *P*2_1_/*n*	Mo *K*α radiation, λ = 0.71073 Å
*a* = 3.9321 (4) Å	Cell parameters from 6357 reflections
*b* = 20.274 (2) Å	θ = 2.9–30.0°
*c* = 8.2685 (9) Å	µ = 7.81 mm^−^^1^
β = 99.602 (2)°	*T* = 90 K
*V* = 649.93 (12) Å^3^	Rod, green
*Z* = 2	1.10 × 0.06 × 0.04 mm

### catena-Poly[[bis(2-methylpyrazine-κN4)copper(II)]-di-µ-bromido] Data collection

**Table:**

Bruker SMART APEX diffractometer	1674 reflections with *I* > 2σ(*I*)
ω scans	*R*_int_ = 0.042
Absorption correction: multi-scan (SADABS; Krause *et al.*, 2015)	θ_max_ = 30.0°, θ_min_ = 2.0°
*T*_min_ = 0.042, *T*_max_ = 0.730	*h* = −5→5
8934 measured reflections	*k* = −28→28
1837 independent reflections	*l* = −11→11

### catena-Poly[[bis(2-methylpyrazine-κN4)copper(II)]-di-µ-bromido] Refinement

**Table:**

Refinement on *F*^2^	Primary atom site location: structure-invariant direct methods
Least-squares matrix: full	Secondary atom site location: difference Fourier map
*R*[*F*^2^ > 2σ(*F*^2^)] = 0.029	Hydrogen site location: inferred from neighbouring sites
*wR*(*F*^2^) = 0.074	H-atom parameters constrained
*S* = 1.08	*w* = 1/[σ^2^(*F*_o_^2^) + (0.0428*P*)^2^ + 0.3*P*] where *P* = (*F*_o_^2^ + 2*F*_c_^2^)/3
1837 reflections	(Δ/σ)_max_ < 0.001
80 parameters	Δρ_max_ = 1.68 e Å^−^^3^
0 restraints	Δρ_min_ = −0.62 e Å^−^^3^

### catena-Poly[[bis(2-methylpyrazine-κN4)copper(II)]-di-µ-bromido] Special details

**Table:**

Refinement. Data were measured using omega scans (0.3 ° per frame for 15 s). All non-hydrogen atoms were refined anisotropically.

### catena-Poly[[bis(2-methylpyrazine-κN4)copper(II)]-di-µ-bromido] Fractional atomic coordinates and isotropic or equivalent isotropic displacement parameters (Å^2^)

**Table:**

	*x*	*y*	*z*	*U*_iso_*/*U*_eq_	
Cu1	0.500000	0.000000	0.500000	0.01973 (12)	
Br1	0.92174 (5)	0.06601 (2)	0.67603 (3)	0.01551 (9)	
N11	0.3718 (5)	0.17387 (10)	0.0988 (2)	0.0173 (4)	
C12	0.2992 (6)	0.18368 (11)	0.2500 (3)	0.0159 (4)	
C13	0.3393 (6)	0.13326 (11)	0.3659 (3)	0.0173 (4)	
H13	0.291403	0.141949	0.472767	0.021*	
N14	0.4438 (6)	0.07281 (9)	0.3308 (2)	0.0167 (4)	
C15	0.5144 (7)	0.06290 (11)	0.1796 (3)	0.0178 (4)	
H15	0.587954	0.020597	0.150055	0.021*	
C16	0.4813 (6)	0.11378 (12)	0.0659 (3)	0.0182 (4)	
H16	0.538781	0.105545	−0.039345	0.022*	
C17	0.1775 (7)	0.25094 (12)	0.2889 (3)	0.0224 (5)	
H17A	0.369251	0.282275	0.296126	0.034*	
H17B	0.093376	0.249652	0.393945	0.034*	
H17C	−0.009587	0.264898	0.202115	0.034*	

### catena-Poly[[bis(2-methylpyrazine-κN4)copper(II)]-di-µ-bromido] Atomic displacement parameters (Å^2^)

**Table:**

	*U*^11^	*U*^22^	*U*^33^	*U*^12^	*U*^13^	*U*^23^
Cu1	0.0253 (2)	0.0148 (2)	0.0164 (2)	−0.00535 (15)	−0.00449 (16)	0.00416 (15)
Br1	0.01469 (13)	0.01564 (13)	0.01582 (14)	−0.00124 (7)	0.00148 (9)	−0.00008 (7)
N11	0.0192 (9)	0.0164 (9)	0.0164 (10)	−0.0007 (7)	0.0035 (8)	0.0013 (7)
C12	0.0153 (10)	0.0153 (10)	0.0168 (11)	−0.0015 (8)	0.0018 (8)	0.0004 (8)
C13	0.0181 (11)	0.0186 (10)	0.0150 (10)	−0.0032 (8)	0.0024 (8)	−0.0007 (8)
N14	0.0183 (10)	0.0161 (9)	0.0144 (10)	−0.0035 (7)	−0.0015 (7)	0.0003 (7)
C15	0.0184 (11)	0.0166 (11)	0.0173 (11)	0.0008 (8)	−0.0003 (8)	−0.0023 (8)
C16	0.0202 (11)	0.0185 (11)	0.0162 (11)	−0.0007 (8)	0.0043 (9)	−0.0008 (8)
C17	0.0267 (12)	0.0175 (11)	0.0238 (13)	0.0025 (9)	0.0062 (10)	−0.0007 (9)

### catena-Poly[[bis(2-methylpyrazine-κN4)copper(II)]-di-µ-bromido] Geometric parameters (Å, º)

**Table:**

Cu1—N14^i^	2.020 (2)	C13—H13	0.9500
Cu1—N14	2.021 (2)	N14—C15	1.340 (3)
Cu1—Br1^i^	2.4196 (3)	C15—C16	1.387 (3)
Cu1—Br1	2.4196 (3)	C15—H15	0.9500
N11—C16	1.335 (3)	C16—H16	0.9500
N11—C12	1.343 (3)	C17—H17A	0.9800
C12—C13	1.392 (3)	C17—H17B	0.9800
C12—C17	1.498 (3)	C17—H17C	0.9800
C13—N14	1.340 (3)		
			
N14^i^—Cu1—N14	180.0	C15—N14—Cu1	121.37 (16)
N14^i^—Cu1—Br1^i^	89.85 (6)	C13—N14—Cu1	121.34 (16)
N14—Cu1—Br1^i^	90.15 (6)	N14—C15—C16	120.7 (2)
N14^i^—Cu1—Br1	90.15 (6)	N14—C15—H15	119.7
N14—Cu1—Br1	89.84 (6)	C16—C15—H15	119.7
Br1^i^—Cu1—Br1	180.0	N11—C16—C15	122.5 (2)
C16—N11—C12	116.8 (2)	N11—C16—H16	118.7
N11—C12—C13	121.0 (2)	C15—C16—H16	118.7
N11—C12—C17	117.4 (2)	C12—C17—H17A	109.5
C13—C12—C17	121.6 (2)	C12—C17—H17B	109.5
N14—C13—C12	121.7 (2)	H17A—C17—H17B	109.5
N14—C13—H13	119.1	C12—C17—H17C	109.5
C12—C13—H13	119.1	H17A—C17—H17C	109.5
C15—N14—C13	117.3 (2)	H17B—C17—H17C	109.5

Symmetry code: (i) −*x*+1, −*y*, −*z*+1.
